# Supplementary material for: Modeling glioblastoma heterogeneity as a dynamic network of cell states
Source: Mol Syst Biol. 2021 Sep 16;17(9):e10105. doi: 10.15252/msb.202010105 (PMC8444284; doi:10.15252/msb.202010105)
Supplement: Supplementary file 6 — Source Data for Figure 5 [file MSB-17-e10105-s004.zip › Figure5A_sourcedata/GSEA_3071/hallmarks_stateI.GseaPreranked.1623742531165/HALLMARK_MYOGENESIS.html]

Details for gene set HALLMARK\_MYOGENESIS[GSEA]

|  || Dataset | state13071 |
| Phenotype | NoPhenotypeAvailable |
| Upregulated in class | na\_pos |
| GeneSet | HALLMARK\_MYOGENESIS |
| Enrichment Score (ES) | 0.42367923 |
| Normalized Enrichment Score (NES) | 2.221477 |
| Nominal p-value | 0.0 |
| FDR q-value | 5.005208E-4 |
| FWER p-Value | 0.002 |
Table: GSEA Results Summary

  

Fig 1: Enrichment plot: HALLMARK\_MYOGENESIS      
 Profile of the Running ES Score & Positions of GeneSet Members on the Rank Ordered List

  

| PROBE | GENE SYMBOL | GENE\_TITLE | RANK IN GENE LIST | RANK METRIC SCORE | RUNNING ES | CORE ENRICHMENT || 1 | COL3A1 |  |  | 3 | 2.646 | 0.0524 | Yes |
| 2 | COL1A1 |  |  | 6 | 2.008 | 0.0923 | Yes |
| 3 | COL6A3 |  |  | 13 | 1.717 | 0.1251 | Yes |
| 4 | SSPN |  |  | 23 | 1.574 | 0.1542 | Yes |
| 5 | COL15A1 |  |  | 25 | 1.550 | 0.1851 | Yes |
| 6 | IGFBP7 |  |  | 45 | 1.387 | 0.2075 | Yes |
| 7 | COL6A2 |  |  | 67 | 1.237 | 0.2264 | Yes |
| 8 | COL4A2 |  |  | 73 | 1.203 | 0.2491 | Yes |
| 9 | APOD |  |  | 76 | 1.195 | 0.2726 | Yes |
| 10 | SPARC |  |  | 106 | 1.067 | 0.2857 | Yes |
| 11 | NQO1 |  |  | 113 | 1.045 | 0.3050 | Yes |
| 12 | SVIL |  |  | 120 | 1.014 | 0.3237 | Yes |
| 13 | BIN1 |  |  | 127 | 0.967 | 0.3414 | Yes |
| 14 | LSP1 |  |  | 143 | 0.930 | 0.3558 | Yes |
| 15 | TGFB1 |  |  | 165 | 0.884 | 0.3675 | Yes |
| 16 | MEF2C |  |  | 210 | 0.804 | 0.3710 | Yes |
| 17 | SOD3 |  |  | 219 | 0.786 | 0.3845 | Yes |
| 18 | AEBP1 |  |  | 236 | 0.761 | 0.3952 | Yes |
| 19 | GSN |  |  | 265 | 0.720 | 0.4016 | Yes |
| 20 | PDE4DIP |  |  | 308 | 0.665 | 0.4028 | Yes |
| 21 | TPM2 |  |  | 320 | 0.655 | 0.4128 | Yes |
| 22 | SH3BGR |  |  | 347 | 0.631 | 0.4179 | Yes |
| 23 | ITGB1 |  |  | 371 | 0.615 | 0.4237 | Yes |
| 24 | LAMA2 |  |  | 506 | 0.503 | 0.3950 | No |
| 25 | DTNA |  |  | 587 | 0.460 | 0.3811 | No |
| 26 | MYH9 |  |  | 701 | 0.411 | 0.3566 | No |
| 27 | BHLHE40 |  |  | 720 | 0.405 | 0.3595 | No |
| 28 | ADAM12 |  |  | 776 | 0.390 | 0.3514 | No |
| 29 | HDAC5 |  |  | 807 | 0.378 | 0.3504 | No |
| 30 | CACNA1H |  |  | 964 | 0.335 | 0.3119 | No |
| 31 | ITGB4 |  |  | 1125 | 0.302 | 0.2716 | No |
| 32 | MYLK |  |  | 1204 | 0.287 | 0.2548 | No |
| 33 | RIT1 |  |  | 1337 | 0.262 | 0.2218 | No |
| 34 | TPM3 |  |  | 1534 | -0.261 | 0.1703 | No |
| 35 | GABARAPL2 |  |  | 1539 | -0.261 | 0.1744 | No |
| 36 | BAG1 |  |  | 1630 | -0.270 | 0.1538 | No |
| 37 | RB1 |  |  | 1771 | -0.290 | 0.1190 | No |
| 38 | NOTCH1 |  |  | 1878 | -0.301 | 0.0944 | No |
| 39 | KCNH2 |  |  | 1937 | -0.311 | 0.0839 | No |
| 40 | SYNGR2 |  |  | 1939 | -0.312 | 0.0898 | No |
| 41 | CHRNB1 |  |  | 1971 | -0.314 | 0.0872 | No |
| 42 | EPHB3 |  |  | 2030 | -0.322 | 0.0769 | No |
| 43 | EFS |  |  | 2038 | -0.323 | 0.0814 | No |
| 44 | CDKN1A |  |  | 2083 | -0.332 | 0.0753 | No |
| 45 | SORBS1 |  |  | 2092 | -0.333 | 0.0797 | No |
| 46 | FST |  |  | 2355 | -0.385 | 0.0115 | No |
| 47 | SORBS3 |  |  | 2428 | -0.398 | -0.0014 | No |
| 48 | MYL6B |  |  | 2767 | -0.494 | -0.0894 | No |
| 49 | APP |  |  | 2770 | -0.495 | -0.0800 | No |
| 50 | MRAS |  |  | 2912 | -0.547 | -0.1098 | No |
| 51 | CHRNA1 |  |  | 2950 | -0.573 | -0.1090 | No |
| 52 | NAV2 |  |  | 2989 | -0.593 | -0.1080 | No |
| 53 | ERBB3 |  |  | 3038 | -0.625 | -0.1094 | No |
| 54 | CDH13 |  |  | 3414 | -1.277 | -0.1923 | No |
| 55 | NCAM1 |  |  | 3416 | -1.284 | -0.1667 | No |
| 56 | PKIA |  |  | 3417 | -1.286 | -0.1408 | No |
| 57 | CLU |  |  | 3424 | -1.329 | -0.1158 | No |
| 58 | ITGA7 |  |  | 3430 | -1.355 | -0.0900 | No |
| 59 | IGFBP3 |  |  | 3433 | -1.384 | -0.0627 | No |
| 60 | CRYAB |  |  | 3475 | -1.829 | -0.0377 | No |
| 61 | CKB |  |  | 3497 | -2.376 | 0.0041 | No |
Table: GSEA details [plain text format]

  

Fig 2: HALLMARK\_MYOGENESIS: Random ES distribution      
 Gene set null distribution of ES for **HALLMARK\_MYOGENESIS**
